# Neural pathways of olfactory kin imprinting and kin recognition in zebrafish

**DOI:** 10.1007/s00441-020-03378-4

**Published:** 2021-01-30

**Authors:** Gabriele Gerlach, Mario F. Wullimann

**Affiliations:** 1 Institute of Biology and Environmental Sciences, Carl-von-Ossietzky University, 26129 Oldenburg, Germany; 2 Helmholtz Institute for Functional Marine Biodiversity Oldenburg (HIFMB), 26129 Oldenburg, Germany; 3 Centre of Excellence for Coral Reef Studies and School of Marine and Tropical Biology, James Cook University, QLD 4811 Townsville, Australia; 4 Graduate School of Systemic Neurosciences & Department Biology II, Ludwig-Maximilians-Universität Munich, 82152 Planegg-Martinsried, Germany; 5 Max-Planck-Institute for Neurobiology, 82152 Planegg-Martinsried, Germany

**Keywords:** Accessory olfactory system, Amygdala, Crypt cells, Imprinting, Kin recognition, Social behavior, Vomeronasal system

## Abstract

Teleost fish exhibit extraordinary cognitive skills that are comparable to those of mammals and birds. Kin recognition based on olfactory and visual imprinting requires neuronal circuits that were assumed to be necessarily dependent on the interaction of mammalian amygdala, hippocampus, and isocortex, the latter being a structure that teleost fish are lacking. We show that teleosts—beyond having a hippocampus and pallial amygdala homolog—also have subpallial amygdalar structures. In particular, we identify the medial amygdala and neural olfactory central circuits related to kin imprinting and kin recognition corresponding to an accessory olfactory system despite the absence of a separate vomeronasal organ.

## Imprinting and kin recognition is widespread

The ability to treat kin differently from non-kin may be achieved by using different mechanisms of kin recognition and is a key driver for kin selection. One type of kin recognition is phenotype matching which describes an individual learning a template of kin and being able to later recognize even unfamiliar kin by matching visual, acoustic, or olfactory components of conspecifics with this template. To reduce potential errors of learning a wrong template this process of learning mostly occurs early in life when the chances to be with relatives is much higher than later when mobility has increased. Such learning within a narrow time window and the often life-long memory is called *kin imprinting*. It is used in a wide context of social behavior to identify the mother in larger groups, aggregate with siblings, cooperate with related conspecifics for hunting or breeding and to avoid inbreeding (Wyatt 2010).

Organisms can also imprint on a variety of non-conspecific (Lorenz 1935) and even abiotic objects (Horn 1998). Take the long-known and spectacularly complex home finding to natal streams of adult salmonids. In the final stage, this behavior at least partially relies on early imprinting on olfactory cues (i.e., free amino acids) of their place of birth (Ueda 2012; 2019; Bett and Hinch 2016; Ueda et al. 2016; Dittman et al. 1996; Kamran et al. 2018; 2019). These early imprinted cues apparently have nothing to do with kin. Another example are marine Anemone fishes that live and breed on coral stocks in close proximity of an anemone. The hatchlings are believed to imprint on chemical cues of the anemone that they encounter during hatching. After an open sea planktonic life stage they can use olfactory cues of an anemone to find a suitable place for settlement (Arvedlund and Nielsen 1996).

There are various examples of kin-biased behavior and recognition among teleosts (for review, see Gerlach and Hinz 2012), mostly revealed by behavioral experiments. A few examples shall illustrate this point: mangrove killifish prefer to associate with their kin (Edenbrow and Croft 2012). Larval guppies (poecilids) can distinguish kin from non-kin (Hain and Neff 2007; Hain et al. 2017) and tend to form shoals with siblings (Piyapong et al. 2011). Closely related mollies (also poecilids) associate with kin using visual and chemical cues (both alone are sufficient) and show aggressive behavior towards non-kin (Makowicz et al. 2016). These three cases represent cyprinodontiforms. Also, salmoniform brook trouts form kin groups early, but dissolve when approaching the breeding stage (Meli and Fraser 2013). Brown trout fry (O’Farrell et al. 2012) and Atlantic salmon juveniles (Rajakaruna et al. 2006) associate with kin as shown by MHC class I or MHC class II allele similarity, respectively. Within perch-like fishes, male adult bluegill sunfishes (*Lepomis macrochirus*) recognize “their” newly hatched offspring using chemical cues (Neff 2003) and their offspring later recognizes kin from non-kin (Hain and Neff 2006). Adult male cichlids (*Pelvicachromis taeniatus*) prefer their own odor over that of other individuals (Thünken et al. 2009) and larva show greater group cohesion with kin than with other individuals, but cohesion decreases with elevated competition (Hesse and Thünken 2014; Thünken et al. 2020). In another cichlid (*Neolamprologus caudopunctatus*), small females tend to form shoals with kin while larger individuals disperse (van Dongen et al. 2014; review on cichlids by Keller-Costa et al. 2015). Adult female sticklebacks differentiate male kin from non-kin based on olfaction alone (Mehlis et al. 2008), whereas in male aggressive interplays, no difference in behavior is apparent towards kin or non-kin males (Mehlis et al. 2009). In some cypriniforms (such as the zebrafish, see below), larval shoaling based on kin recognition is present. Kin recognition-based group association has been suggested recently in two coral reef living species of damselfish (percomorph pomacentrids) (Miller-Sims et al. 2008; Buston et al. 2009; review Gerlach et al. 2019). Regarding the special case of kin recognition in the context of shoaling, an important issue is to test whether experimental results mimic naturally occurring kin groupings (Krause et al. 2000).

In any case, these examples from taxa as diverse as salmoniforms, cypriniforms, gasterosteiforms, cyprinodontiforms or cichliforms and other percomorphs are clear evidence that kin recognition is widespread among freshwater and maybe marine teleosts. However, although it is likely that this “knowledge” about kin relationship arises during early life history through a process of imprinting, details on the involved peripheral sensory mechanism, let alone on the central neural underpinnings of imprinting and kin recognition are notoriously evasive in all these cases. In contrast, the beauty of the zebrafish example is that we have a good understanding of the entire life-history of the imprinting process and the later behavioral and neural outcome.

## Zebrafish kin related behavior and olfactory periphery

### Zebrafish kin imprinting and kin recognition

Research in zebrafish represents one of the best-defined and most coherently documented cases of imprinting on kin and kin recognition in vertebrates. This work ranges from behavioral experiments, molecular genetics, immunohistochemical identification of underlying neural structures at peripheral and central nervous levels, neuronal tract-tracing of pathways involved, as well as experimental activation of neuronal structures along these pathways using the neuronal activity related marker phosphorylated extracellular signal regulated kinase (pERK).

The behavioral aspects of kin imprinting, the general olfactory epithelial sensory cell composition, their receptor molecules and—if known—ligands, their differential primary projections to the olfactory bulb, as well as the analysis of which sensory cells and olfactory glomeruli are involved in kin imprinting and recognition have been reviewed recently by Gerlach and colleagues (Gerlach et al. 2019). Thus, these topics will be only briefly introduced here.

Depending on temperature (25°C), zebrafish larvae hatch from the chorion at day 4 dph and 5 dph (days posthatching). Shortly thereafter, the imprinting process starts while larvae are only starting to be mobile. Larvae that were exposed to the visual cues of siblings at 5 dph and at 6 dph to olfactory cues of siblings that shared the same MHC class II alleles became imprinted on siblings. An olfactory preference for siblings is the result when zebrafish were still sexually immature juveniles, but as adults, females avoided the scent of brothers and males were interested in olfactory and visual cues of females, but did not care about relatedness (Gerlach and Lysiak 2006). These time windows for imprinting on visual and olfactory cues are short and last only for 1 to 2 days (Gerlach et al. 2008). If imprinting does not occur during this period, zebrafish do not express any kin recognition later on. Interestingly, larvae only imprint on kin but not on non-kin cues; when they were exposed to visual or olfactory cues of non-kin during this sensitive phase, they did not imprint and do not show any preference for the experienced non-kin (Hinz et al. 2013a). The only exception occurs when non-kin share the same MHC class II alleles with the zebrafish larvae. In this case, zebrafish imprinted on MHC class II identical zebrafish non-kin larvae and showed a preference for kin later in life (Hinz et al. 2013b). To understand the chemical composition of the olfactory cues, we tested larvae from different zebrafish pairs and MHC class II alleles for their response on MHC ligands which have been shown to influence olfactory choice in sticklebacks beforehand. In one of these “family” groups of larvae, we observed a significant olfactory preference for the MHC ligand mix consisting of 5 different MHC ligands. Surprisingly, by adding these ligands at 6 dph, we could also trigger an olfactory preference for kin when larvae had had visual contact to kin at 5 dph. We concluded that this mix of MHC ligands was the olfactory cue used for imprinting. These results of behavioral choice tests and screens for identifying MHC similarity asked for a closer look at the neuronal processes that happen at 6 dph when olfactory imprinting takes place. Based on RT-qPCR or microarrays, the expression of olfactory receptor genes did not differ in imprinted and non-imprinted fish (Gerlach et al. 2019). Thus, we concluded that perhaps different from salmons (Dittman et al. 2015; Madsen et al. 2019), imprinting does not change the frequency or composition of olfactory receptors in the nasal epithelium, but other mechanisms must be responsible. Therefore, we will have a look at the peripheral level of olfactory perception.

### Zebrafish olfactory periphery

**Fig. 1:**
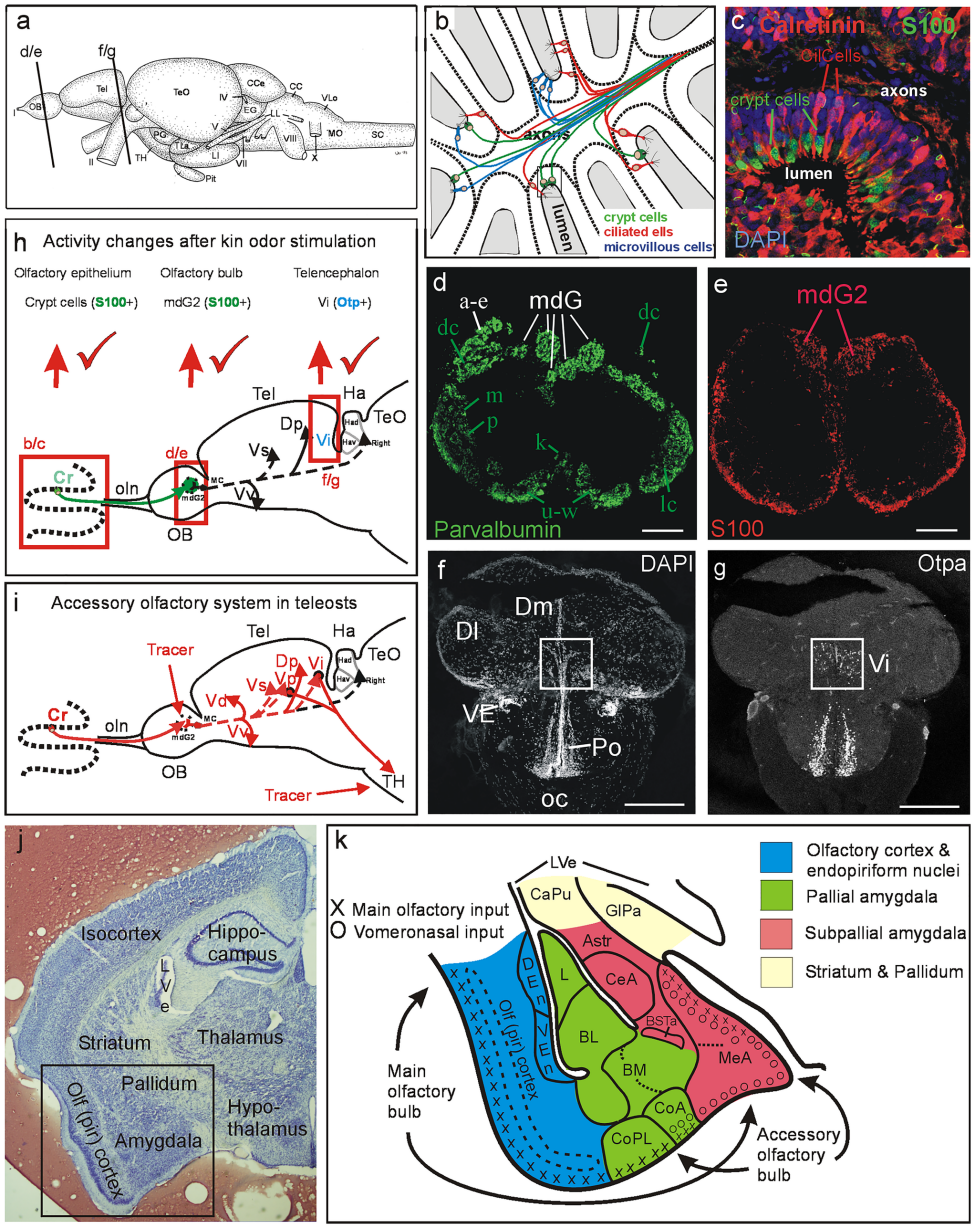
Teleostean and mammalian amygdala and accessory olfactory system. **a** Lateral view of adult zebrafish (*Danio rerio*) brain with indication of section levels shown for olfactory bulb (**d/e**) and telencephalon (**f/g**). **b** Schema shows the three main classes of teleostean/zebrafish olfactory sensory neurons (OSNs). Crypt cells, as all other OSN types, are widely distributed over the entire olfactory epithelium (modified after Kress et al. 2015). **c** Double-label immunohistochemistry for calcium-binding proteins in adult zebrafish olfactory epithelium demonstrates that calretinin (as also calbindin1 and parvalbumin, both not shown here) is absent in crypt cells which are solely characterized by S100 (modified after Kress et al. 2015). **d**, **e** Transverse sections through adult zebrafish olfactory bulb show parvalbumin (**d**) and S100 (**e**) immunostained fibers (modified after Kress et al. 2015; see there for details). Lower key green letters in **d** designate groups of olfactory bulb glomeruli, white letters designate calretinin/calbindin1-free glomerular groups, e.g., the mediodorsal bulbar glomeruli. Apparent parvalbumin fibers in mdG2 originate from a subpopulation of microvillous OSNs, not from crypt cells. **e** S100 fibers from crypt cells converge into one glomerulus, i.e., mdG2. Red elements at bulbar periphery are glial. **f**, **g** Neuroanatomical identification of the intermediate nucleus of ventral telencephalon (Vi) as the homolog of the medial amygdala using nuclear stain DAPI (**f**) and immunohistochemistry for Otpa (**g**) (modified after Biechl et al. 2017). **h**, **i** Schematics of primary and secondary olfactory pathways in adult zebrafish (modified after Biechl et al. 2017). **h** Neuronal activity as quantified with pERK at three successive synaptic levels from peripheral OSNs in the olfactory epithelium to two consecutive central nervous targets, including the olfactory bulb glomerulus mdG2 (immunohistochemically identified with S100 antibody because the projections of S100 immunopositive crypt cells terminate there; see text and **e**) and the intermediate nucleus of the telencephalon Vi (immunohistochemically identified with Otpa antibody for many of its cell bodies, see text and **g**) after kin odor stimulation of kin-imprinted and non-imprinted larvae. The counted pERK-positive cells were located in the olfactory epithelium, around the mdG2 and within Vi. Red tickmarks indicate statistically significant changes in activated cell numbers seen at each level (see Biechl et al. 2017 and text for details). Adult secondary olfactory projections of mediodorsal bulbar area are indicated with solid black lines (targets shared with projections of entire olfactory bulb) and dashed black lines (targets specifically attributed to mediodorsal bulbar area; see literature below). **i** Projections of adult zebrafish mediodorsal olfactory bulb area (including mdG2) shown after lipophilic tracing substance DiI injection (Biechl et al. 2017). Additional tracer injections into tuberal hypothalamus (TH) demonstrate furthermore a teleostean accessory olfactory pathway via subpallial amygdalar nuclei Vp/Vi. Olfactory bulb projections shown as dashed lines to telencephalic targets are selective for mediodorsal olfactory bulb (our data are shown in red lines, confirmed and extended by additional data from Ahuja et al. 2013; Kress et al. 2015; Sato et al. 2005; Braubach et al. 2012; Miyasaka et al. 2009; Gayoso et al. 2012; Turner et al. 2016). **j** Transverse Nissl-stained frontal section through left mouse forebrain (*Mus musculus*, section by courtesy of Dr. Alex Kaiser) with major telencephalic and diencephalic regions indicated. Frame indicates region shown schematically in **k**. **k** Mouse amygdala and olfactory (piriform) cortex. Blue: olfactory (piriform) cortex and endopiriform nuclei. Green: pallial amygdala (lateral, basolateral, basomedial, and cortical amygdala). Red: subpallial amygdala (amygdalo-striatal transition area, central, and medial amygdala, intra-amygdalar bed nucleus of stria terminalis) (redrawn and simplified after Martínez-García et al. 2012). Main olfactory epithelium input reaches via main olfactory bulb the piriform cortex and most (pallial) cortical amygdalar nuclei (but not the large posteromedial nucleus, not shown in **k**) as well as the dorsal part of the (subpallial) medial amygdala. Vomeronasal organ input reaches via accessory olfactory bulb the pallial part of the anterior amygdala (not shown), the entire medial amygdala and part of the cortical amygdala (its anterior nucleus, CoA, and, in particular, its large posteromedial nucleus (not shown). No vomeronasal input reaches the posterolateral cortical amygdalar nucleus, CoPL. Abbreviations: AStr, Amygdalo-striatal transition area; BL, Basolateral (or basal) amygdala; BM, Basomedial (or accessory basal) amygdala; BSTa, Intra-amygdalar part of bed nucleus of the stria terminalis; CaPu, Caudate-putamen (striatum); CC, Crista cerebellaris; CCe, Corpus cerebelli; CeA, Central amygdala; CilCells, Ciliated cells (olfactory sensory neurons); CoA, Anterior part of cortical amygdala; CoPL, Posterolateral part of cortical amygdala; Cr, Crypt cells; DEn, Dorsal endopiriform nucleus; Dl, Lateral zone of dorsal telencephalic area; Dm, Medial zone of dorsal telencephalic area; Dp, Posterior zone of dorsal telencephalic area; EG, Eminentia granularis; GlPa, Globus pallidus; Ha, Habenula; Had, Dorsal part of Ha; Hav, Ventral part of Ha; L, Lateral amygdala; LI, Hypothalamic lobus inferior; LL, Lateral line nerves; LVe, Lateral ventricle; mdG, Mediodorsal group of olfactory bulb glomeruli; mdG2, Mediodorsal olfactory bulb glomerulus 2; MeA, Medial amygdala; MO, Medulla oblongata; MS, Medulla spinalis; oc, Optic chiasma; OB, Olfactory bulb; oc, Optic chiasma; OE, Olfactory epithelium; Olf (pir) cortex, Olfactory (piriform) cortex; oln, Olfactory nerve; Pit, Pituitary; Po, Preoptic region; SC, Spinal cord; Tel, Telencephalon; TeO, Optic tectum; TH, Tuberal hypothalamus; TLa, Torus lateralis; Vd, Vi, Vp, Vs, Vv, Dorsal, intermediate, postcommissural, supracommissural, ventral nucleus of ventral telencephalic area; VEn Ventral endopiriform nucleus; VE, ventral entopeduncular nucleus; VLo/LX, Vagal lobe. Cranial nerves: I Olfactory nerve, II Optic nerve, IV Trochlear nerve, VII Facial nerve, VIII Octaval nerve, X Vagal nerve

Olfactory chemoreception is dependent on the binding of an odorant to its corresponding receptors located on microvilli or cilia of olfactory sensory neurons (OSNs), with subsequent signal transmission to the central nervous system. Various types of OSNs are embedded in the olfactory epithelium (Fig. 1b; for recent reviews, see Olivares and Schmachtenberg 2019; Gerlach et al. 2019).

Two major populations of OSNs are present in teleosts—ciliated OSNs (cOSNs) and microvillous OSNs (mOSNs)—resembling OSNs present in tetrapod main and vomeronasal olfactory systems, respectively (Fig. 1b, c; reviewed by Eisthen 1997; Korsching 2016; Gerlach et al. 2019). The cOSNs have deeply located cell nuclei in the olfactory epithelium and long dendrites with apical cilia as well as an axon on the opposite side. In zebrafish, cOSNs terminate profusely in the dorsolateral and ventromedial part of the olfactory bulb (Kress et al. 2015). Microvillous OSNs (mOSNs) have usually less deeply located cell nuclei and therefore shorter dendrites than cOSNs, and they have microvilli extending from their apical surface and an axon on the other cell side (Yamamoto and Ueda 1979; Thommesen 1983; Hansen and Finger 2000; Kress et al. 2015). The zebrafish olfactory bulb targets of mOSNs are some dorsal, in particular mediodorsal, and ventrolateral glomeruli (Kress et al. 2015). Some (and maybe all) teleosts feature two more OSN types: crypt cells (Hansen and Finger 2000; Ahuja et al. 2013; Kress et al. 2015), which are spherical or pear-shaped cells located close to the olfactory epithelial surface (crypt cells are equipped with both a few cilia and microvilli), and Kappe cells which have microvilli (Ahuja et al. 2014). Both are named after their peculiar cellular morphology. Kappe cells are only known in teleosts, but crypt cells occur in teleost and cartilaginous fish (Ferrando et al. 2006). Interestingly, these two OSN cell types project with their axons to only one glomerulus each in the mediodorsal olfactory bulb, i.e., crypt cells to mdG2 (Fig. 1e) and Kappe cells to mdG5 (reviewed in Gerlach et al. 2019).

We will have a closer look at what is known about crypt cells, their expressed olfactory receptor, the potential role of crypt cells in triggering social behavior and their response to kin odor. Oka and colleagues (Oka et al. 2012) showed that the olfactory receptor ora4 belonging to the v1r-like ora genes is expressed in all crypt cells whereas the remaining five ora genes were not found in this cell type. However, this receptor alone cannot explain the differential responses to kin and non-kin. More receptors have to be involved to explain the remarkable abilities to differentiate between many different MHC ligands. It is still not clear whether MHC class II similarity between recipient and signaler changes the intensity of ligand binding. A ligand–MHC protein–odorant receptor interaction perhaps evoked by odorant-binding proteins could enhance the solubility of hydrophobic odors and facilitate the transport of odors to receptor sites (Pelosi 1994) and might lead to a stronger neuronal activation compared with that in larvae that are exposed to peptide ligands of non-kin odor. In addition to crypt cells, we showed that a small subpopulation of mOSNs responded to kin odor. Considering that MHC peptides are a component of kin odor, these mOSNs might express a V2R receptor and bind to such MHC peptides. This is also consistent with the fact that our MHC-related GCaMP2 activation was seen in the dorsolateral olfactory bulb and not in the mediodorsal bulb area (reviewed in Gerlach et al. 2019). The nature of the kin signal processed in crypt cells remains elusive.

While the total number of crypt cells did not differ in imprinted versus non-imprinted zebrafish larvae, a significantly higher number of crypt cells was activated after kin odor stimulation in imprinted compared with non-imprinted larvae and compared with imprinted control larvae stimulation in our pERK activity assay (Biechl et al. 2016). No difference in activation was found within non-imprinted larvae. As the observed higher neuronal activity in specific OSNs (crypt cells and a subpopulation of mOSNs) in imprinted larvae did not correspond with an increased number of these OSNs, olfactory imprinting might be explained by a change in binding sensitivity of the odorant receptor itself. Another mechanism leading to differential neuronal activity might be based on inhibition. Oka et al. (2004) showed in mice that odorants can inhibit odorant responses of olfactory receptors (ORs), which is evidence of antagonism between odorants at the receptor level. Behavioral and psychophysical studies demonstrated that mixing some odorants led to the emergence of novel perceptual qualities that were not present in each component, suggesting that odorant mixture interactions occurred at some levels in the olfactory system (Jinks and Laing 2001; Wiltrout et al. 2003). Thus far, the ligand that binds to ora4 is still unknown. However, based on our data, the ligand of ora4 is contained in kin odor, but it is unlikely that ora4 is the specific receptor for MHC peptides in zebrafish (Boschat et al. 2002; Isogai et al. 2011).

In crucian carp, *Carassius carassius*, crypt cells have been found to respond to sex pheromones and differed in numbers according to the reproductive season (Hamdani et al. 2008). During winter, few crypt cells were observed at any location within the sensory epithelium. In spring, the majority of crypt cells were located deep in the epithelium not yet exposed to the environment. However, during the summer spawning season, crypt cells were positioned at the epithelial surface. Quantification of the density and relative position of crypt cells in the lamellae of the common carp revealed that their density increases significantly with sexual maturity in both males and females (Adair et al. 2018). This example of a similar biological role of crypt cells in a closely related (cyprinid) species compared to zebrafish demonstrates that crypt cells might be adapted to slightly different contexts in the socio-sexual realm in teleosts.

## Central pathways for kin imprinting and kin recognition in zebrafish

The presence of various olfactory sensory neuron (OSN) types, their morphological and molecular characterization, and differential projection patterns to the olfactory bulb have been summarized above for the zebrafish. The central olfactory pathways shall be analyzed next by particularly focussing on zebrafish brain regions that have a role in social contexts such as kin recognition. A usual suspect in all things concerning social interactions in vertebrates is the accessory olfactory system that involves in amniotes a pathway from the vomeronasal organ via an (accessory) part of the olfactory bulb to the medial amygdala, and from there to the medial hypothalamus (see below) and we will discuss its identification in the zebrafish.

### The comparative context: the mammalian amygdala and telencephalon

*Development and neuroanatomy*. In order to understand the comparative significance of recent neurobiological results on kin imprinting and kin recognition in the zebrafish, the general neurobiological context of the mammalian amygdala needs some consideration. The mammalian amygdala—although small in comparison to cortex and basal ganglia (see mouse brain section shown in Fig. 1j)—is increasingly recognized as an agglomeration of various subnuclei (Fig. 1k). These have complex patterns of inputs and intrinsic circuitry, as well as outputs devoted to different functions (Pitkänen et al. 1997; Swanson and Petrovich 1998, Martínez-García et al. 2008; 2009a, b; 2012; Tovote et al. 2015). The rodent amygdala comprises more than twenty nuclei and these are of two embryonic origins: pallial (cortex-like; green in Fig. 1k) or subpallial (basal ganglia-like; red in Fig. 1k).

An important mammalian pallial amygdalar complex is formed by three nuclei which are the lateral (L), basolateral (= basal) (BL, with three divisions), and basomedial (= accessory basal) (BM; with three divisions) nuclei (Fig. 1k), summarily also called basolateral amygdala or complex (Martínez-García et al. 2009a). This large part of the pallial amygdala arises embryonically from the most ventral pallial division and, thus, produces excitatory glutamatergic cells. Also all remaining pallial divisions are characterized by autochthonous generation of glutamatergic cells, i.e. the lateral (olfactory or piriform cortex), the medial pallium (hippocampus) and the dorsal pallium (isocortex). For a recent discussion of lateral versus ventral pallial amygdalar origin, see Wullimann (2017).

A second major part of the pallial amygdala comprises the cortical amygdala (CoA, CoPL; Fig. 1k; CoPM, not shown). Similar to the olfactory (or piriform) cortex (Fig. 1k), the anterior (CoA) and posterolateral (CoPL) cortical amygdalar nuclei receive sensory input from the main olfactory epithelium via the main olfactory bulb (unlike the remaining pallial amygdala, e.g., the CoPM, L, BL, BM).

In contrast, the central amygdala (CeA; Fig. 1k) is subpallial in nature. Thus, it arises embryonically from the caudal ganglionic eminence (sometimes considered the most caudal divisions of both lateral and medial ganglionic eminences; García-López et al. 2008) and, as a result, the central amygdala consists mostly of inhibitory GABAergic neurons. The latter also applies to the main part of the subpallium, the basal ganglia (caudate-putamen and globus pallidus; CaPu/GlPa, Fig. 1k) which arise from lateral and medial ganglionic eminences, respectively. The central amygdala is free of any olfactory bulb input.

A second major part of the mammalian subpallial amygdala is the medial amygdala. Its entire superficial surface receives sensory vomeronasal organ input via the accessory olfactory bulb (Fig. 1k). Moreover, only the dorsal part of medial amygdala is joined by main olfactory epithelial input via main olfactory bulb. In turn, vomeronasal input joins main olfactory epithelium input in (pallial) anterior cortical amygdala (CoA) and the small (subpallial) bed nucleus of the accessory olfactory tract (BAOT), and is even the sole olfactory input to the cortical amygdalar posteromedial nucleus (CoPM) (the latter two nuclei not shown in Fig. 1k). Another (non-olfactory) part of the subpallial amygdala is the (central and medial) extended amygdala which includes in addition to its small intra-amygdalar part (BSTa; Fig. 1k) a series of nuclei (bed nuclei of stria terminalis (BNST)) extending anteriorly towards the vicinity of the anterior commissure (Martínez-García et al. 2012).

*Amygdalar connectivity and function*. The basolateral amygdalar complex has input and output connections with sensory thalamus and isocortex/hippocampus (Doron and Ledoux 1999; 2000; Martínez-García et al. 2009a; 2012) which provides for ongoing (thalamus) and highly processed multisensory (isocortex) as well as declarative memory (hippocampus) information. The basolateral complex has a critical role in conditioned fear learning and fear memory (LeDoux 2000; Tovote et al. 2015). Its behavioral output (via anterior BM + anterior and posterior BL) is mediated via central amygdala to lateral hypothalamus and various brainstem centers (e.g., dorsal vagal complex and periaqueductal gray) regulating fear and anxiety related bodily (autonomic) and behavioral (motor) reactions, respectively (Martínez-García et al. 2009a; 2012). Important for autonomic responses is that the central amygdala receives also ascending visceroceptive/gustatory inputs from brainstem (parabrachial and solitary tract nuclei) and thalamus. Minor direct isocortical and hippocampal inputs to the central amygdala also exist.

A second main output system arises in both basolateral complex (anterior and posterior BM) and cortical amygdala (CoA, CoPL, CoPM; the latter three nuclei are all dominated by olfactory input; see above) and runs via medial amygdala to medial hypothalamus (including preoptic region) and septum to guide socio-sexual and defensive behaviors (Martínez-García et al. 2009a; 2012). In contrast to older views, also this system involving the medial amygdala includes effects on autonomic, in addition to behavioral, responses. Amygdalar pathways to medial hypothalamus represent the stria terminalis; those to lateral hypothalamus are called ansa lenticularis. The various bed nuclei of stria terminalis (BNST; see above) are bidirectionally interconnected with either central or medial amygdala (therefore together with them called the extended amygdala) and contribute efferent axons to hypothalamic and other forebrain targets of the medial amygdala or to long descending connections of the central amygdala (Martínez-García et al. 2009a; 2012).

The pathway from vomeronasal organ, via accessory olfactory bulb and medial amygdala to medial hypothalamus is also referred to as the accessory olfactory system, as opposed to the main olfactory system running from main olfactory epithelium via main olfactory bulb to olfactory cortex (Martínez-García et al. 2009b). As can be judged from the foregoing, this is a highly simplified concept. In reality, the cross-connectivity between main olfactory and vomeronasal organ central connections is of stunning complexity (Martínez-García et al. 2009a; 2012).

The cortical amygdala (in addition to the subpallial medial amygdala) provides a strong pallial olfactory connotation to the socio-sexual/defensive system, but lacks largely isocortical interconnections, though it has a substantial output (of CoPM) to the hippocampus. The basolateral (BM, BL) and cortical amygdala (CoPM) further project to striatum in order to provide for reward and motivational behavioral contexts in motor output. Thus, through highly complex intrinsic and extrinsic interconnectivity, the amygdalar outputs finally lead into hypothalamus and brainstem guiding appropriate motor, endocrine and autonomic processes related to emotional behaviors (fear, anxiety, aversion vs reward, motivation, attraction) (Pitkänen et al. 1997; Martínez-García et al. 2009b, 2012; Tovote et al. 2015).

*Comparative search for amygdalar subdivisions*. In an admirably deep going discussion of molecular genetic, neurochemical, developmental and functional data, Martínez-García and colleagues (2009a) provided a well supported proposal of homologies of all amygdalar subdivisions in land vertebrates (tetrapods) and, in particular, among amniotes (i.e., mammals, birds, reptiles). However, the understanding of the amygdala in bony and cartilaginous fishes remained elusive for longer. This changed with elegant experiments involving discrete pallial lesions and behavioral testing in goldfish by Cosme Salas and Fernando Rodríguez and their colleagues. This work established that teleostean pallial medial and lateral divisions (Dm and Dl; Fig. 1f) have functions highly similar to the amniote pallial amygdala (Dm, highly reminiscent of mammalian basolateral complex and its role in fear learning; Portavella et al. 2002; 2004) and hippocampus (Dl, related to place memory; Rodríguez et al. 2002; et al. Salas et al. 2003; Bandoh et al. 2011). Importantly, these behavioral associations of Dm and Dl fit generally the long known phenomenon of pallial eversion in teleosts (Wullimann 2009; see below).

The recognition of Dm as the teleostean pallial amygdala fostered a preoccupation with it, but also a neglect of the subpallial amygdala in fish. Thus, many confirmatory studies on the teleostean pallial amygdala (Dm) followed (e.g., Lau et al. 2011; von Trotha et al. 2014; Ruhl et al. 2015; Silva et al. 2015; Lal et al. 2018). In contrast, the teleostean medial amygdala remained elusive (e.g. Perathoner et al. 2016) or was even concluded to be absent in teleosts. In the latter view, the teleostean postcommissural subpallium (Vs, Vp; see below) corresponds entirely to mammalian central amygdala and BNST (Ganz et al. 2012; Maximino et al. 2013).

**Fig. 2:**
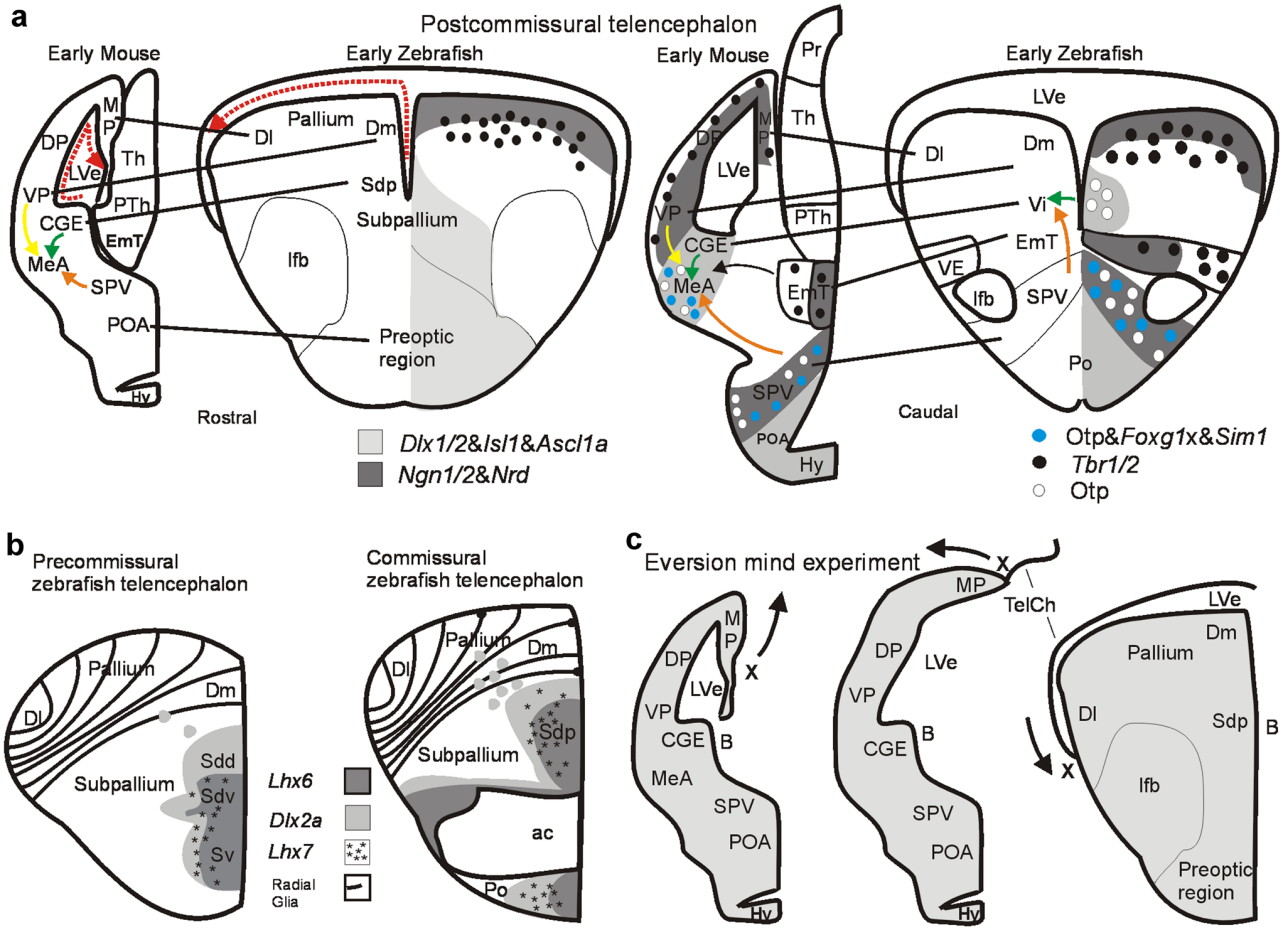
Development of the amygdala in early mouse and zebrafish brain. **a** Rostral (left panel) and caudal (right panel) transverse sections of postcommissural telencephalic mouse and zebrafish brains with critical pallial and subpallial gene markers indicated. Gene expression for mouse after Osório et al. 2010; Lo et al. 1991; Ma et al. 1997; Horton et al. 1999; Torii et al. 1999; Fode et al. 2000; Puelles et al. 2000), Morales et al. 2020; for zebrafish after Mueller et al. 2008; Herget et al. 2014, Affaticati et al. 2015; Mueller and Wullimann 2016 (see there for details). Dotted red arrows indicate topological lateromedial course of pallial ventricular surface. Solid yellow arrow indicates tangential migration of glutamatergic ventral pallial cells into mouse medial amygdala. Solid orange arrow indicates tangential migration of Otp positive glutamatergic cells from hypothalamic supraopto-paraventricular region (SPV) into medial amygdala (MeA; mouse)/intermediate nucleus of ventral telencephalon (Vi; zebrafish). Solid green arrow indicates radial migration of subpallial GABAergic cells into medial amygdala (mouse)/intermediate nucleus of ventral telencephalon (Vi, zebrafish). **b** Subpallial gene expression in early precommissural and commissural zebrafish telencephalon identifies within the larval subpallium a ventral division (Sv; adult ventral nucleus of ventral telencephalon, Vv, the septum homolog) and a dorsal division (Sd, adult dorsal nucleus of ventral telencephalon, Vd, the basal ganglia homolog). Sd is further genetically subdivided into ventral (Sdv) and dorsal (Sdd) subdivisions, representing the pallidal and striatal homologs, respectively (and embryonic mammalian medial and lateral ganglionic eminences). At the (commissural) level of the anterior commissure, the posterior division of the larval zebrafish dorsal subpallial division (Sdp) starts. In the adult zebrafish brain, it represents at this level the supracommissural nucleus of the ventral telencephalon (Vs). Panels in **b** modified after Mueller et al. (2008). The larval Sdp continues into postcommissural levels (seen in **a** left panel), represented in the adult zebrafish brain by the postcommissural nucleus of the ventral telencephalon (Vp). Only recently, a most posterior ventral telencephalic (subpallial) area characterized by otp positive cells was recognized (Herget et al. 2014; Biechl et al. 2017) and identified as the intermediate nucleus of the ventral telencephalon (Vi) in larval and adult zebrafish brains (see **a**, right panel, and Fig. 1). Note that the course of radial glia fibers is indicated in the zebrafish pallium. **c** This mind experiment illustrates how a postcommissural early rodent telencephalon is transformed into a postcommissural early zebrafish telencephalon by everting pallial masses. The most medial rodent pallial division (MP/hippocampus; note position of letter X and arrow indicating eversion) is virtually rolled out laterally resulting in the topography of the postcommissural teleostean/zebrafish telencephalon by maintaining topological relationships of major telencephalic divisions (see text). Compare a similar figure in Wullimann (2009) for the precommissural telencephalon. Abbreviations: ac, anterior commissure; CGE, caudal ganglion eminence; Dl, lateral zone of dorsal telencephalic area; Dm, medial zone of dorsal telencephalic area; DP, dorsal pallium; EmT, eminentia thalami; Hy, hypothalamus; lfb, lateral forebrain bundle; LVe, lateral ventricle; MeA, medial amygdala; MP, medial pallium; Po, preoptic area (zebrafish); POA, anterior preoptic area (mouse); PTh, prethalamus; Sd larval dorsal part of subpallium; Sdd, dorsal subdivision of Sd (striatum homomog); Sdv, ventral subdivision of Sd (pallidum homolog), Sdp, posterior subdivision of Sd (subpallial amygdala homolog); SPV, supraopto-paraventricular region; Sv, larval ventral part of subpallium; TelCh, tela chorioidea, Th, (dorsal) thalamus; VE, ventral entopeduncular nucleus; Vi, intermediate nucleus of ventral telencephalon; VP, ventral pallium

In order to trace the teleostean medial amygdala, the postcommissural (i.e., lying posterior to the anterior commissure) rodent telencephalon first needs consideration (Fig. 2a; two levels are shown). At these levels, the caudal ganglion eminence (CGE) is seen to provide by radial migration GABAergic cells to the subpallial amygdala (shown is the medial amygdala; MeA; Bupesh et al. 2011a, b; Abellán et al. 2013; Morales et al. 2020). These GABAergic cells are necessarily characterized by expression of specific transcription factors, such as Dlx1/2, Isl1, and Ascl1a, which are typical of the entire subpallium and most of hypothalamus (Fig. 2a; see legend for citations for gene expression). However, also tangential migration of inhibitory GABAergic cells from subpallial to pallial areas is common. The most extensive example is that of cortical GABAergic interneurons, all of which arise from the embryonic (subpallial) ganglionic eminences (Marín and Rubenstein 2001). Similarly, the basolateral amygdalar complex comprises 20% GABAergic neurons (Tovote et al. 2015), presumably having tangentially migrated there from the subpallial ganglionic eminences.

In turn, excitatory glutamatergic cells migrate tangentially into the mouse medial amygdala from nearby brain structures, such as ventral pallium (VP), the hypothalamic supraopto-paraventricular region (SPV), and the eminentia thalami (EmT) (García-Moreno et al. 2010; Bupesh et al. 2011a, b; Abellán et al. 2013; Morales et al. 2020). Cells in these three regions all share gene expression typical also of pallial (cortical) areas, such as *Ngn1/2,Nrd* (genes that are essential in glutamatergic neuronal development), and neuronal differentiation genes, such as *Tbr1/2*, shared by cortex and eminentia thalami (Fig. 2a). The glutamatergic cells in medial amygdala that arise from the supraopto-paraventricular area SPV are selectively characterized by expression of *Orthopedia (Otp) *(Fig. 2a). Furthermore, *Pax6* expressing medial amygdalar cells arising by tangential migration from the eminentia thalami (EmT) were described in the mouse (Bupesh et al. 2011b). All these glutamatergic cellular contributions to the medial amygdala arise through tangential migration, whereas medial amygdalar GABA cells from the caudal ganglionic eminence arrive by radial migration (Bupesh et al. 2011a). This fact identifies the medial amygdala principally as subpallium because radial migration is decisive for identifying brain divisions as seen in the case of massive tangential invasion of subpallial cells into mammalian cortex (see above and Wullimann 2020). Furthermore, the mouse medial amygdala has two different populations of *Otp* cells arising in SPV, one without and one with additional expression of transcription factors *Foxg1/Sim1* (Fig. 2a). Thus, the mammalian/rodent medial amygdala is a mosaic of GABAergic subpallial cells complemented by glutamatergic neuron types from extrinsic sources (ventral pallium, SPV, EmT).

### The teleostean amygdala and telencephalon

We will now discuss the zebrafish medial amygdala. As mentioned above, the recognition of Dm as the teleostean pallial amygdala was paralled by a lack of convincing arguments for teleostean subpallial amygdalar divisions. The obvious start of this discussion is at the olfactory periphery because (main and vomeronasal) olfactory input characterizes the medial amygdala in land vertebrates.

*Main and vomeronasal olfactory epithelium*. Most amniotes, and to a certain degree amphibians (Syed et al. 2017), usually have a separate main olfactory epithelium (MOE) and a vomeronasal organ (VNO). Notably, absence or reduction of a VNO is seen in humans and other primates, as well as in bats and whales (Eisthen 1992). The respective central pathways of MOE and VNO are well segregated at least up to the olfactory bulb and partly beyond it (see above details for rodents; Fig. 1k). The two main pathways lead firstly from the MOE via main olfactory bulb mostly into the olfactory cortex (lateral pallium) and cortical amygdala and, secondly, the VNO projects via accessory olfactory bulb (AOB) mostly to the medial amygdala and from there to the medial hypothalamus. These pathways are addressed as the main and accessory olfactory systems, respectively (see above).

How to discern these two pathways in teleost fishes where a separate MOE and VNO are never discernible? As discussed above (see “Zebrafish kin related behavior and olfactory periphery” section) both microvillous and ciliated OSNs exist together with additional OSN types (Kappe cells, crypt cells) widely scattered over one large main olfactory epithelium and are not segregated into vomeronasal (microvillous OSNs) and main olfactory (cililated OSNs) epithelia or organs. Teleostean olfactory bulb projections reach all subpallial subdivisions and, in the pallium, the posterior zone of the dorsal telencephalon (Dp) and part of the most ventral lateral zone (reviewed in Wullimann and Mueller 2004; Vernier and Wullimann 2009; Fig. 1i). We will not discuss the pallial olfactory connections here, but rather focus on the subpallium and the question whether there is a teleostean medial amygdala and a functional accessory olfactory system. Before that, a short overview on the teleostean telencephalon seems appropriate.

*The zebrafish telencephalon*. How does the teleostean/zebrafish telencephalon relate in comparative terms to the amniote/mammalian one? The teleostean precommissural (i.e., in front of the anterior commissure) nuclei of the ventral telencephalic area (subpallium) include the adult ventral (Vv) and dorsal nucleus (Vd) (corresponding to larval ventral/dorsal part of subpallium, Sv/Sd; Fig. 2b). These were long suspected to correspond to septal and striatal divisions of the subpallium, respectively (review by Northcutt and Braford 1980). Eventually, the adult zebrafish Vd (= larval Sd) was shown to receive ascending input (Rink and Wullimann 2001) from dopaminergic posterior tubercular cells corresponding to the anterior part of the vertebrate mesodiencephalic dopamine cell complex, an input typical of the amniote striatum (reviewed in Wullimann and Umeasalugo 2020). The adult Vv projects to midline hypothalamus typical of the amniote septum (Rink and Wullimann 2004).

Furthermore, molecular genetic data (differential expression of *Lhx6/7*) showed that the zebrafish Sd can be subdivided into a ventrally located larval pallidum (Sdv) and striatum (Sdd) dorsally to it (Fig. 2b left panel; discussed in Mueller et al. 2008). The posterior continuation of the larval subpallium at commissural (Sdp; Fig. 2b right panel) and postcommissural telencephalic levels (Sdp; Fig. 2a) corresponds in the adult brain to the supracommissural (Vs) and postcommissural (Vp) nuclei of the ventral telencephalic area, respectively. Thus, it had been hypothesized for a long time that Vs/Vp correspond to the teleostean subpallial amygdala (i.e., the central and medial amygdala) generally for teleosts (Northcutt and Braford 1980) and for zebrafish in particular (Mueller et al. 2008). However, more refined arguments for either central or medial amygdala in teleosts remained elusive as mentioned already.

Recent work on the expression of *orthopedia* (*otp*) revealed in the zebrafish larval and adult hypothalamic preoptic area the supraoptic-paraventricular region (SPV) previously seen in other vertebrates (Herget et al. 2014; Affaticati et al. 2015) (see above). The SPV represents the core of the hypothalamic stress axis containing numerous types of neuropeptidergic neurons (paraventricular nucleus in amniotes corresponds to magnocellular preoptic nucleus in teleosts) and the teleostean SPV is also characterized by different gene expression than the surrounding hypothalamus (Herget et al. 2014; Affaticati et al. 2015), as similarly discussed above for the mouse (Fig. 2a). Many Otp-(protein) positive cells migrate from this hypothalamic preoptic expression site seemingly tangentially into the most posterior ventral zebrafish telencephalon (Fig. 1g). The zebrafish SVP furthermore has Otp-positive cells that co-express *Foxg1/Sim1* (Affaticati et al. 2015) and, presumably, such cells also migrate into the telencephalon as in the mouse (see above).

There is a general consensus that the pallial masses in teleosts are everted, i.e. the teleostean medial pallium (hippocampus homolog) therefore comes to lie laterally (Dl), whereas the ventral pallium (pallial amygdala homolog) remains medially (Dm; Fig. 2a, the red dotted arrows indicate the ventricular surface from ventral into medial pallium and they run in reversed direction due to eversion). The mind experiment shown in Fig. 2c illustrates the eversion process in the postcommissural telencephalon by virtually transforming the early rodent telencephalon into a postcommissural early zebrafish telencephalon. If one takes the medial beginning of the rodent medial pallium (point X) and virtually everts it laterally (note black arrows in Fig. 2c), the pallial masses end up in the topography of a teleostean telencephalon. Hereby topological relationships of pallial masses are maintained. Note in particular that the caudal ganglion eminence conforms topologically to the posterior division of the dorsal subpallium (Sdp) which fits its identification as medial amygdala. A similar figure can be found for the precommissural telencephalon in Wullimann (2009).

*The search for the zebrafish medial amygdala and the accessory olfactory system*. We then investigated the telencephalic Otp-positive cells in more detail in the adult zebrafish and identified them as being in the intermediate nucleus of the ventral telencephalon (Vi; Fig. 1f, g; Biechl et al. 2017) because Otp-positive cells seem to qualify as diagnostic for the vertebrate medial amygdala (as discussed above for mammals). The Vi had been described by Levine and Dethier (1985) as one of various olfactory bulb projection targets in the goldfish and was recognized in this same position in channel catfish (Bass 1981). Various studies in zebrafish have established that the dorsomedial olfactory bulb area receives exclusively microvillous OSN and crypt cell input (Gayoso et al. 2012; Braubach et al. 2012; Ahuja et al. 2013; Kress et al. 2015). Furthermore, only one single mediodorsal glomerulus (mdG2) receives S100-positive axons from all crypt cells and from a subpopulation of microvillous OSNs, the latter are at the same time parvalbumin positive (Kress et al. 2015; Biechl et al. 2017). Moreover, crypt cells (and a subpopulation of S100-negative microvillous OSNs) are selectively activated by kin odor in imprinted zebrafish larvae in activation experiments involving pERK as a read-out (Fig. 1h; see below and Biechl et al. 2016). This connectional and functional information on a specific pathway from microvillous/crypt cells to a special (i.e., dorsomedial) division of the zebrafish olfactory bulb conforms to the first synaptic step of an accessory olfactory (“vomeronasal”) system.

Next, the higher order connections were studied by putting neuronal tracer DiI into the zebrafish mediodorsal bulb area (Biechl et al. 2017; Fig. 1i). In addition to central projections common to the entire olfactory bulb (solid red lines to Vd/Vv/Dp in Fig. 1i), some are specific to the mediodorsal olfactory bulb (dashed lines to Vs/Vp, and Vi). Thus, it appears that the entire subpallial zebrafish telencephalon receives secondary olfactory projections. Importantly, those to Vi are from the mediodorsal olfactory bulb and represent the second synaptic step of an accessory olfactory system. We then administered DiI to the medial tuberal hypothalamus and found retrograde label in Vi (and Vp) (Fig. 1i; Biechl et al. 2017). This is the third and final synaptic step or requirement for an accessory olfactory system.

Parallel neuronal activation experiments investigated olfactory bulb cells surrounding the only glomerulus (mdG2, recognizable in pERK stainings through S100 double-label, Fig. 1e) that receives S100 positive crypt cell/microvillous OSN input. Indeed, olfactory bulb cells around mdG2 were activated by kin odor in imprinted larvae in significantly higher numbers than in non-imprinted larvae (Biechl et al. 2017). Finally, a significant difference was also found for pERK activated Otp-positive cell numbers in the posterior telencephalic subpallial area that was newly identified intermediate nucleus of the ventral telencephalon (Vi; hypothesized above as the medial amygdala homolog already). However, the difference was between non-imprinted fish (higher activated cell numbers) compared to imprinted fish and control groups (lower activated cell numbers). Possibly, non-imprinted fish show a neuronal response in Vi (medial amygdala) to the unknown kin odor whereas this response is alleviated or lost in imprinted fish (Gerlach et al. 2019).

Finally, a comprehensive study on the entire amygdaloid complex of zebrafish recently provided the most detailed basis to date for a functional neuroanatomical and developmental understanding of the teleostean amygdala and telencephalon in an explicit comparative context with the mammalian (mouse) situation (Porter and Mueller 2020). The richness of data and arguments in this study far exceed a thorough discussion here. However, the main advances regarding the amygdaloid complex are as follows. The zebrafish medial amygdala is confirmed to include the Otp-positive Vi described above, but additionally has Otp-negative subdivisions within Vs and Vp as well as in a most dorsal subdivision of Vd (Porter and Mueller 2020). The zebrafish central amygdala has several subdivisions, such as the subpallial central nucleus (Vc) and two divisions in parts of Vd dorsal to the striatal one (Porter and Mueller 2020). Zebrafish bed nuclei of the stria terminalis are identified in the telencephalic strands of dopamine cells along the various subpallial nuclei and in part of Vs (Porter and Mueller 2020). Interestingly, the cortical amygdala is identified within Dm and the majority of Dm is confirmed as the remaining pallial amygdala (Porter and Mueller 2020). This paper represents clearly a milestone in comparative teleostean forebrain research because it solves long-standing questions of homology between mammalian and teleostean telencephala and therefore long-term pressing phylogenetic problems. However, some interpretations based on developmental arguments may need reconsideration in the light of radial versus tantential migration. From our foregoing discussion it is clear that we do not consider the Vi homologous to part of the eminentia thalami as Porter and Mueller (2020) do. The Vi may receive cellular contributions from EmT by tangential migration as is reported for the rodent medial amygdala (see discussion above). However, similarly we do also not see Vi as homologous to the hypothalamic SPV (from which the medial amygdala/Vi receives a substantial amount of otp-positive cell numbers in mouse and zebrafish). We rather interpret these two non-telencephalic areas as contributors of cells to medial amygdala/Vi by tangential migration, whereas the decisive radial contribution of GABA cells to medial amygdala/Vi is subpallial and identifies it as a part of subpallial amygdala (see Wullimann 2020 for a discussion of the general problem).

In summary, the general position of Vi within the subpallial telencephalon, a “vomeronasal” type olfactory input (crypt/microvillous OSNs) via dorsomedial olfactory bulb and activity differences between imprinted and non-imprinted zebrafish larvae along this neuronal pathway (Fig. 1h), as well as efferent projections of Vi to the medial hypothalamus (Fig. 1i) are convincing arguments for identifying Vi as a part of the teleostean medial amygdala. To our knowledge this was the first time that neuroanatomical, developmental and functional data were presented together in a teleost to identify an accessory olfactory pathway associated to a socio-sexual context.
